# Challenging of treating patients with exfolliative cheilittis: Report of two cases

**DOI:** 10.4317/jced.60326

**Published:** 2023-05-01

**Authors:** Lílian-Rocha Santos, Thaylla-Núñez-Amin Dick, Monique-Santana Candreva, Letícia-Côgo Marques, Adrianna Milagres, Rafaela-Elvira Rozza-de-Menezes, Arley-Silva Junior, Karin-Soares Cunha, Danielle-Castex Conde

**Affiliations:** 1 DDS, MSc. Postgraduate Program in Pathology, School of Medicine, Universidade Federal Fluminense (UFF), Niterói, RJ, Brazil; 2 DDS. Postgraduate Program in Pathology, School of Medicine, Universidade Federal Fluminense (UFF), Niterói, RJ, Brazil; 3 DDS, PhD. Department of Pathology, School of Medicine, Universidade Federal Fluminense (UFF), Niterói, RJ, Brazil; 4 DDS, PhD. Postgraduate Program in Pathology, School of Medicine, Universidade Federal Fluminense (UFF), Niterói, RJ, Brazil

## Abstract

Cheilitis is a term given to the inflammation that occurs in the vermillion of the lips. The exfoliative type is an uncommon form of cheilitis, which is characterized by inflammation and desquamation of the lip. It can cause aesthetic problems and compromise daily eating and phonation. The aim of this paper is to describe two cases of exfoliative cheilitis in young persons under periods of emotional stress and parafunctional habits. A 22-year-old white male and an 18-year-old black female presenting edema, intense dryness, and slight desquamation on the vermilion of the lips. In the second case, fissures with bleeding were also observed. Oral lesions were associated with intense emotional stress. The diagnosis of both was made based on the clinical presentation and the exclusion of other conditions. Although the patients have presented a significant improvement after the corticosteroid treatment, they still have a recurrence in stressful episodes. Detailed clinical examination and complementary exams are fundamental for determining associated factors and correctly diagnosing exfoliative cheilitis. Treatment can be challenging, especially in the face of relapses.

** Key words:**Cheilitis, exfoliative cheilitis, oral lesions, stress psychological.

## Introduction

Cheilitis is a term given to the inflammation that occurs in the vermilion of the lips or the transition zone between the skin and the labial mucosa (1,2). It can manifest in several ways; the most common types are angular, actinic, allergic contact cheilitis, plasmacytic, glandular, granulomatous, factitious, and exfoliative (2,3). The exfoliative type is an uncommon form characterized by inflammation and desquamation of the lip, which can cause aesthetic problems and compromise daily activities, such as eating and phonation (1).

Exfoliative cheilitis consists of excessive production of keratin in the vermilion of the lips with persistent fissures, dryness, and scaling that commonly involves the upper and lower lips (2). The etiology of exfoliative cheilitis remains unknown. It may occur as an isolated condition or as part of certain systemic diseases (2). It is more common in young people, mainly women, who have a lip-licking habit (2). An association with vitamin B12 or iron deficiency, poor oral hygiene, oral candidiasis (cheilocandidiasis), psychiatric problems, and a candidiasis variant of the acquired human immunodeficiency syndrome (HIV) has also been reported (2,4,5). Anxiety and emotional stress have been linked as causes and/or exacerbators of this condition (6). This paper aims to describe two cases of exfoliative cheilitis in young persons under periods of emotional stress who also had parafunctional habits.

## Case Report

The two patients described here were attended at the Oral Diagnosis Outpatient Clinic of the Antônio Pedro University Hospital of the Fluminense Federal University (HUAP), Niterói, RJ, Brazil, and signed an informed consent form.

Case 1 

**Figure 1:**
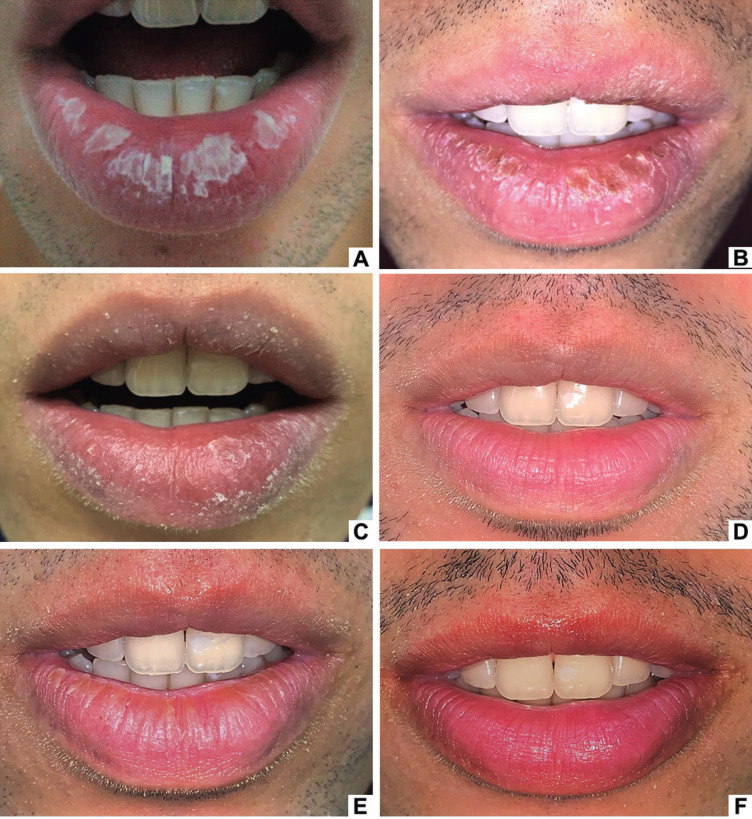
Clinical aspects of Case 1. White plaques on the lower lip (A). Yellowish-white crusts on the upper and lower lips (B). Improvement of the clinical appearance after using topical corticosteroids (C). Complete resolution of the lesions on the upper and lower lips after using corticosteroids (D). Recurrence of the lesions: yellowish crusts and dryness of the lower lip (E). Remission of lesions on the lips after a new drug intervention (F).

A 22-year-old white male patient was seen at the HUAP dermatology outpatient clinic complaining of “burning and peeling lips” (Fig. 1A). On the physical examination, the vermillion of the lips had edema, intense dryness, and slight desquamation. The diagnosis of contact cheilitis was initially established, and the replacement of the toothpaste with another containing natural and herbal agents was indicated. However, there was no improvement in the lesions. A lip moisturizer (Eucerin Aquaphor® – panthenol, bisabolol, and glycerin) was then prescribed, and a partial improvement of the dryness and scaling of the lips was observed after three weeks. However, edema and the burning symptoms persisted. The patient was then referred to the Oral Diagnosis clinic for evaluation and treatment.

During the anamnesis, the patient reported that the lesions appeared four months earlier, during vacation, after intense sun exposure without protection, with exacerbation and partial remission episodes. Two months after the appearance of the first lesions, he noticed a worsening of desquamation and symptoms after an anxiety crisis. He also stated that there were no changes in his routine, denied the application of cosmetic products during this period, and reported the habit of lip licking. Past medical histories included asthma, respiratory allergies, and anxiety disorder. He had the habit of smoking tobacco and marijuana and using alcoholic beverages. Multiple white-yellowish crusts were observed on the entire extension of the lower lip vermilion and in the central region of the upper lip vermilion on physical examination (Fig. 1B).

Unstimulated whole saliva (UWS) sialometry was within a normal range (1.1 mL/min), and the cytopathological examination showed mild inflammation and ruled out candidiasis. Laboratory tests were negative for syphilis (Venereal Disease Research Laboratory, VDRL), HIV (anti-HIV 1 and 2), hepatitis B (HBsAg), and hepatitis C (anti-HCV). The blood count, coagulogram, serum levels of vitamin B12 and iron had no alterations.

**Figure 2:**
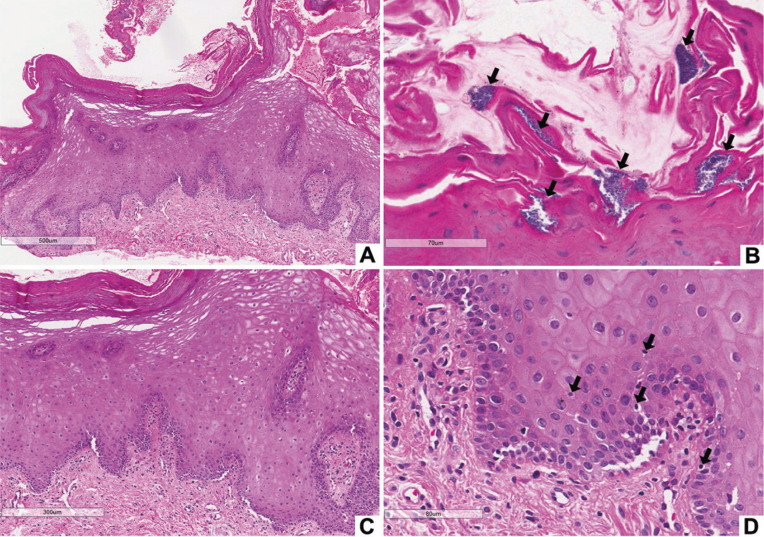
Histopathological aspects of Case 1. Stratified squamous epithelium with hyperparakeratinization (A). Note bacterial colonies on the epithelial surface (B, black arrows). Presence of acanthosis and cellular edema (C), mild lymphocyte exocytosis (black arrows), and scarce subepithelial chronic inflammatory infiltrate (D).

An incisional biopsy of the lower lip was performed, and the histopathological examination revealed a parakeratinized squamous epithelium with acanthosis, cellular edema, and mild lymphocyte exocytosis. Bacterial colonies were noticed on the epithelium surface. The underlying connective tissue had a mild inflammatory infiltrate, predominantly lymphocytic (Fig. 2). Based on the clinical presentation, the clinical history, and other complementary tests, the diagnosis of exfoliative cheilitis was established.

The proposed treatment included three topical corticosteroids of different potency, which were carried out in three stages in a weekly therapeutic regimen. Therapy started with a high-potency corticosteroid (clobetasol propionate, 0.5 mg every six hours) in the first week. In the second week, clobetasol propionate was used every eight hours. In the third week, clobetasol was used every 12 hours; and in the fourth week, every 24 hours. After this, a medium-potency topical corticosteroid (Omcilon-A M: 1.0 mg of triamcinolone acetonide, 2.5 mg of neomycin base, 0.25 mg of gramicidin, and 100,000 IU of nystatin) was prescribed in the same application scheme described earlier. Lastly, a low-potency topical corticosteroid (1% hydrocortisone acetate) was prescribed with the same application scheme indicated in the first and second stages. Moreover, lip moisturizer with sun protection factor (Hidradeep profuse® FPS 30) was maintained on demand. Toothpaste without sodium lauryl sulfate was also prescribed. The patient was instructed to stop the habit of lip licking and to avoid smoking tobacco and marijuana. He was referred for psychiatric treatment and began using benzodiazepines (chloridrate of sertraline 500mg/day and clonazepam 0.5mg/day).

In the first week of treatment, a significant improvement in edema, desquamation, and dryness was observed (Fig. 1C). After three weeks of treatment, there was a complete remission of the lesions and symptoms. (Fig. 1D). However, two months after finishing the treatment, tenuous yellowish crusts and dryness appeared on the lower lip after a new anxiety crisis, requiring a new intervention with the same therapeutic scheme (Fig. 1E). After finishing the treatment, there was a complete remission of the lesions (Fig. 1F). For two and a half years, the patient was monitored. Relapses occurred during stressful episodes when the habit of lip-licking became more intense.

Case 2 

**Figure 3:**
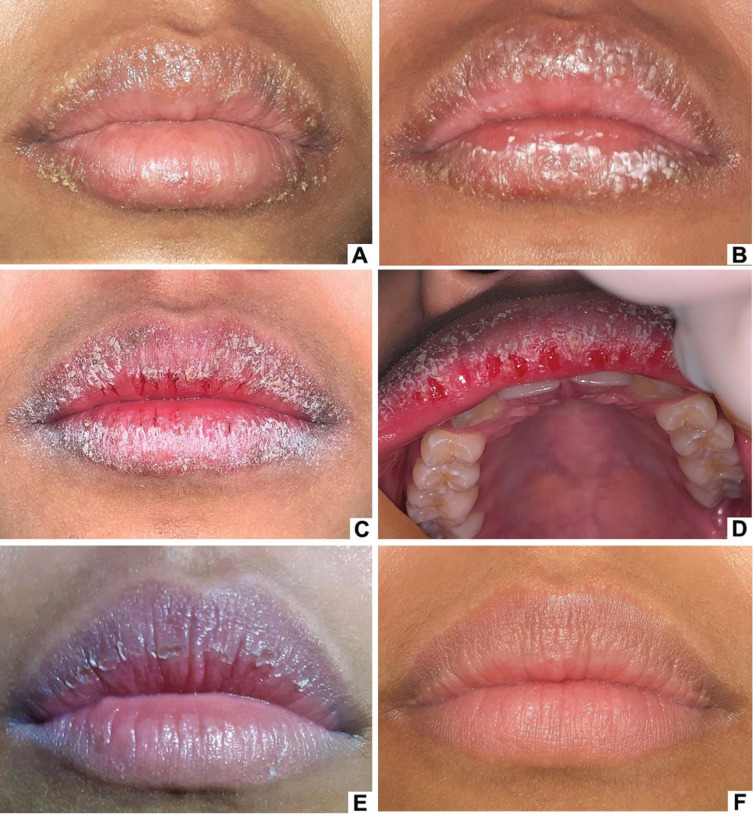
Clinical aspects of Case 2. Vesicles and white-yellowish crusts on the upper and lower lips (A). Evolution of the lesion with white plaques on the lower lip (B). Presence of desquamation, fissures, and bleeding in the vermilion of the upper lip (C and D) and upper lip (D). Improvement of the clinical aspect after using topical corticosteroid (E). Lips with no lesion after a year and a half of follow-up. (F).

An 18-year-old black female patient was seen at the medical outpatient clinic due to the presence of vesicles on the lips that began to appear after intense emotional stress that occurred 15 days ago and evolved to desquamation (Fig. 3A,B). The medical team established the initial diagnosis of recurrent herpes simplex infection and prescribed systemic and topical acyclovir (the medical team did not report the scheme) for 15 days. However, there was no improvement. Thus, 20 mg of oral prednisone were prescribed for five days, with partial improvement in the lesions. Nevertheless, after discontinuing the medication, the lesions worsened, with occurrence of desquamation and cracking of the lips. The patient was then referred to the outpatient oral diagnosis clinic.

During the anamnesis, stressful episodes were reported without follow-up or a medical diagnosis. She denied addictive habits, changes in routine, or the use of cosmetics in the region. During the consultation, the habit of lip-licking was observed. On physical examination, there was intense dryness, desquamation, fissures, and bleeding in the vermilion of the upper and lower lips that limited mouth opening (Figs. 3C,D). Although the patient no longer had vesicles, she did not tolerate manipulation of the lips due to pain and intense discomfort. Therefore, material collection for cytopathological analysis could not be performed. UWS sialometry was within the normal range (0.3 mL/min). Laboratory tests were negative for VDRL syphilis, HIV-1 and HIV-2, HBsAg, and anti-HCV, and the blood count, coagulogram, and serum levels of vitamin B12 and iron were within normal levels.

The proposed treatment included Omcilon-AM for four weeks in the same scheme described in Case 1. A lip moisturizer with sun protection factor (Nivea sun® FPS 30) was prescribed on demand between topical corticosteroid applications. The patient was instructed to interrupt the habit of lip-licking and was referred for psychotherapy; however, she has not sought psychological treatment. One month after beginning the treatment, the patient reported complete remission of the desquamation, fissures, bleeding, and dryness of the lips. Currently, the patient has been followed-up for a year and a half at the oral diagnosis outpatient clinic, presenting relapses during episodes of anxiety.

## Discussion

Although the etiology of exfoliative cheilitis is unknown, one of the main hypotheses is that its development is due to parafunctional activities such as lip-licking (6,7). Saliva contains digestive enzymes that are harmful to the lips’ protective barrier, promoting dryness, desquamation, and fissures (8). Lip-licking is commonly associated with anxiety and depression, which occurred in the current cases, but it is also associated with climate change, chronic dryness (history of atopic dermatitis), mouth breathing, and some systemic diseases, such as Sjögren’s syndrome, and medication use (8).

The onset of exfoliative cheilitis is commonly associated with emotional stress (1,9), and psychological and psychiatric disorders may contribute to developing and worsening this condition (7,9). In a series of 48 cases of exfoliative cheilitis, 87% had psychiatric disorders (10). Patients treated with antidepressants usually show improvement, although there is no complete remission of the lesions (1). Both patients in the present report mentioned emotional stress and anxiety as initiating or aggravating factors of the condition. Moreover, they also had the habit of lip-licking. The first patient associated this parafunctional habit with periods of greater emotional stress and reported improvement with antidepressant medication. The second patient was unable to observe such a relationship.

The first patient reported that the onset of lesions occurred after intense sun exposure. It is believed that with excessive exposure to the sun, cold, and climate changes, the lips become more dry, leading to lip-licking (8). This habit momentarily relieves the symptoms but can perpetuate the condition (8). Other factors, such as vitamin and mineral deficiency (vitamin B12 or iron), bacterial and fungal infections, and smoking, have been associated with this type of cheilitis (11). Furthermore, in a study of patients with AIDS, a prevalence of 28.5% of exfoliative cheilitis was found, and some cases had a candida infection association, which may be a spectrum of candidiasis in these patients (5). In the cases presented, laboratory tests (VDRL, HIV, HBsAg, anti-HCV, blood count, coagulogram, vitamin B12, and iron) showed no changes. In case 1, exfoliative cytology ruled out candidiasis. In contrast, in case 2, it was not possible to collect material because the patient had a symptom of pain at the time of consultation. The oral hygiene of both was satisfactory. 

Exfoliative cheilitis is characterized by a cyclic mechanism of thickening the superficial layers of keratin, which come off at different rates and in various locations on the lip. Fissures, edema, and bleeding may be present. Formation of crusts is common, and they tend to peel off, leaving a bloody surface, with subsequent formation of new crusts (11). The most commonly reported symptoms are burning, itching, sensitivity, and pain (11). In Case 1, the patient had multiple white-yellowish crusts on the upper and lower lips. However, unlike the typical clinical manifestations of exfoliative cheilitis, the vermilion surface was not bloody after desquamation. Thus, contact stomatitis was considered a clinical diagnostic hypothesis. Case 2, on the other hand, was quite characteristic, with intense dryness, desquamation, fissures, and bleeding in the upper and lower vermilion, as well as mouth-opening limitation and pain. Furthermore, confirming the data in the literature (6), both patients complained about aesthetic problems.

Anamnesis and the clinical appearance of the lesions are frequently sufficient for the diagnosis of exfoliative cheilitis (1,12,13). However, in atypical cases, as in Case 1, clinicopathological correlations are indicated to exclude other illnesses. Histopathological features usually include parakeratosis and inflammation (8). Depending on the stage of evolution of the lesion, the degrees of keratosis, fibrosis, epithelial hyperplasia, and inflammation can vary; however, those histopathological findings are nonspecific (14). Fungal or bacterial colonies are usually present on the surface of the epithelium and are probably secondary findings (6,7). The presence of bacterial colonies on the surface of the epithelium was observed in Case 1.

The differential diagnosis of exfoliative cheilitis includes cheilocandidiasis and actinic, glandular, allergic contact, and factitial cheilitis (12). Despite the report of the occurrence of lesions after intense exposure to the sun, in Case 1, no clinical and histopathological characteristics compatible with actinic cheilitis were observed, such as epithelium atrophy and solar elastosis. Glandular cheilitis was ruled out due to the absence of salivary glands with chronic inflammation. Cytopathological examination ruled out the presence of fungi. Furthermore, patients denied using cosmetics on the lips. However, to help exclude allergic contact cheilitis, toothpaste with a natural formulation, without sodium lauryl sulfate, was prescribed. After two months, no improvement was observed in the clinical conditions of either patient.

The literature reports that exfoliative cheilitis treatment is a challenge due to the varied responses of patients to the protocols used (15). Although some cases are resolved, at least temporarily, some others persist for years (4), characterizing a chronic disease (6). There are few studies available on this topic, and the use of topical and systemic corticosteroids, antibiotics, keratolytic products, herbal medicines, salicylic acid ointments, tacrolimus, cryotherapy with liquid nitrogen, and laser therapy are some of the therapeutic options mentioned for the treatment of exfoliative cheilitis (15-17). The association of steroids, psychotherapy, and tranquilizers tends to present a more predicTable response (12). However, few studies describe the therapeutic limitations of topical and systemic steroids in the treatment of exfoliative cheilitis (9).

Given the available therapeutic options, in Case 1, we opted for the prescription of three topical corticosteroids of different potencies (low, medium, and high), with weekly changes in dosage, aiming at reducing signs and symptoms as well as decreasing the probability of relapses. Based on the literature, it was associated with psychotherapeutic treatment. In Case 2, topical therapy with medium-potency corticosteroids was prescribed for four weeks, with resolution of the condition.

Exfoliative cheilitis, a diagnosis of exclusion, is characterized by persistent keratotic and desquamative vermilion of the lips. Detailed clinical examination and complementary exams are fundamental for determining the associated factors and establishing the correct diagnosis. Treatment can be challenging, especially in the face of relapses. The multidisciplinary team is essential for accompanying patients with exfoliative cheilitis.
